# Down-Regulation of NF-κB Target Genes by the AP-1 and STAT Complex during the Innate Immune Response in *Drosophila*

**DOI:** 10.1371/journal.pbio.0050238

**Published:** 2007-09-04

**Authors:** Lark Kyun Kim, Un Yung Choi, Hwan Sung Cho, Jung Seon Lee, Wook-bin Lee, Jihyun Kim, Kyoungsuk Jeong, Jaewon Shim, Jeongsil Kim-Ha, Young-Joon Kim

**Affiliations:** 1 Department of Biochemistry, National Creative Research Initiative Center for Genome Regulation, Yonsei University, Seoul, Korea; 2 Department of Molecular Biology, Sejong University, Seoul, Korea; Stanford University, United States of America

## Abstract

The activation of several transcription factors is required for the elimination of infectious pathogens via the innate immune response. The transcription factors NF-κB, AP-1, and STAT play major roles in the synthesis of immune effector molecules during innate immune responses. However, the fact that these immune responses can have cytotoxic effects requires their tight regulation to achieve restricted and transient activation, and mis-regulation of the damping process has pathological consequences. Here we show that AP-1 and STAT are themselves the major inhibitors responsible for damping NF-κB–mediated transcriptional activation during the innate immune response in *Drosophila*. As the levels of dAP-1 and Stat92E increase due to continuous immune signaling, they play a repressive role by forming a repressosome complex with the *Drosophila* HMG protein, Dsp1. The dAP-1–, Stat92E-, and Dsp1-containing complexes replace Relish at the promoters of diverse immune effector genes by binding to evolutionarily conserved *cis*-elements, and they recruit histone deacetylase to inhibit transcription. Reduction by mutation of dAP-1, Stat92E, or Dsp1 results in hyperactivation of Relish target genes and reduces the viability of bacterially infected flies despite more efficient pathogen clearance. These defects are rescued by reducing the Relish copy number, thus confirming that mis-regulation of Relish, not inadequate activation of dAP-1, Stat92E, or Dsp1 target genes, is responsible for the reduced survival of the mutants. We conclude that an inhibitory effect of AP-1 and STAT on NF-κB is required for properly balanced immune responses and appears to be evolutionarily conserved.

## Author Summary

The immune response is designed to target foreign infectious elements, not self, but it can become destructive when it fails to discriminate self from nonself. Therefore, it is important to restrain the magnitude and duration of the immune response by several mechanisms including receptor down-regulation and inhibitor synthesis. Here, focusing on the immune system of *Drosophila*, we present a mechanism of control that relies on the transcription factors AP-1 and STAT to prevent the excessive activation of the NF-κB–mediated immune response. Thus, AP-1 and STAT, renowned for their role in activating the NF-κB–mediated immune response, appear also to participate in its attenuation. In their role as negative regulators, AP-1 and STAT form a complex with HMG protein and HDAC. This complex is then recruited to the promoter regions of NF-κB target genes, causing the chromatin structure near the NF-κB target genes to contract and the expression of NF-κB target genes to shut down. Mis-regulation of this negative-feedback process, we found, increased the lethality of bacterial infection in *Drosophila*. A similar scenario has been noted in mammals with over-activated NF-κB–mediated immune responses, which has been implicated in autoimmune disease. Thus, feedback inhibition of NF-κB appears to be evolutionarily conserved to maintain properly balanced immune responses.

## Abstract 3

After a role in initiating an NF-κB-mediated innate immune response to microbial challenge, AP-1 and STAT act to form part of a repressosome to down-regulate the transcription of antimicrobial peptides and thus to resolve the immune response.

## Introduction

The innate immune response triggered by pathogen infection activates signal transduction pathways and elicits diverse humoral and cellular responses [1,2]. This response requires the activation of several transcription factors to remodel the gene expression pattern of cells [3]. NF-κB plays a key role in the synthesis of antimicrobial peptides and cytokines [4,5], whereas the transcription factors AP-1 and STAT regulate genes involved in phagocytosis and melanization [6–8]. Nuclear receptors and SMAD proteins also influence the expression of inflammatory cytokines [9,10]. In many cases, the functions of these transcription factors do not appear to be limited to the synthesis of particular effector molecules, but also regulate the activities of other transcription factors involved in such biological processes as immune responses [11,12]. For example, AP-1 transcription factors are reported to interact with the histone deacetylase complex [13] and to suppress Smad2 transcription [14]. Stat1 is required for the interferon-γ suppression of c-*myc* expression in mouse embryo fibroblasts [15]. In addition, NF-κB–dependent Fas transcription is down-regulated by the suppressive action of c-Jun and STAT3 in human melanoma-derived cell lines [16], and NF-κB bound to the *Il6* and *Il12b* promoters is gradually replaced by ATF3, which interacts with AP-1 and STAT [17]. These interactions play key roles in the proper maintenance and termination of immune responses. However, the precise nature of the positive or negative cross-talk between these transcription factors is still unclear, as is the physiological role of such cross-talk in the innate immune response. To address these issues, we examined positive and negative interactions between transcription factors during the response to lipopolysaccharide/peptidoglycan (LPS/PGN). We found that two transcription factors, dAP-1 and Stat92E, which are activated by LPS/PGN-induced signal transduction pathways, form a repressosome complex together with the *Drosophila* HMG protein, Dsp1 (dorsal switch protein) and histone deacetylase, and this then inhibits transcription of diverse immune effector genes activated by Relish. We also found that mis-regulation of negative cross-talk increased the lethality of bacterial infection in *Drosophila*, as has been noted in mammals with over-activated NF-κB–mediated immune responses. Therefore, the inhibitory effect of this repressosome complex on NF-κB plays an important role in maintaining properly balanced immune responses and appears to be evolutionarily conserved.

## Results

### Requirement for Stat92E and Jra for Down-Regulating Relish Target Genes

**Figure 1:**
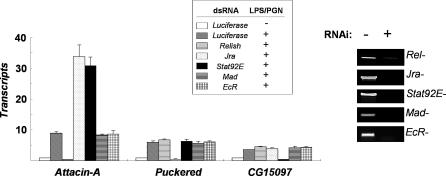
Down-Regulation of Relish Signaling by Stat92E as well as Jra in Response to LPS/PGN Real-time PCR analysis showing LPS/PGN-induced transcriptional activation in various mutant backgrounds. SL2 cells were incubated with dsRNA, as indicated in the top box, for three days. The levels of the transcripts before (-) and after (+) LPS/PGN treatment (10 μg/ml; 1hr) were measured by real time PCR. The degree of depletion of the corresponding transcript by RNAi is shown in the right panel.

To test for the presence of regulatory cross-talk between the signaling pathways of innate immunity, we examined the involvement of key transcription factors (Relish, Jra, Stat92E, Mad, and EcR) in LPS/PGN-induced immune responses in *Drosophila* SL2 cells (Figure 1). To this end, we knocked down each transcription factor by RNA interference (RNAi), and examined its effect on the LPS/PGN-induced transcriptional activation of *Attacin-A, Puckered,* and *CG15097*, the known targets of transcription factors Relish, dAP-1 and Stat92E, respectively. LPS/PGN-induced transcriptional activation of *Attacin-A, Puckered,* and *CG15097* was abolished only by depletion of the corresponding transcription factor, and no obvious transcriptional defect was observed as a result of depletion of Mad or EcR (the *Drosophila* SMAD and nuclear receptor, respectively). Intriguingly, Relish-dependent transcriptional activation of *Attacin-A* was hyperactivated in the absence of Jra or Stat92E. The repressive effect of Stat92E on Relish-dependent transcription required the activated form of Stat92E, because knock-down of *Hopscotch* resulted in an increase of LPS/PGN-induced *Attacin-A* expression comparable to that in the Stat92E-depleted cells (Figure S1). Therefore, the Relish-dependent transcriptional activation of *Attacin-A* appears to be down-regulated by activated Stat92E as well as Jra during the innate immune response.

### Recruitment of Stat92E to the Relish-Dependent Promoter

**Figure 2:**
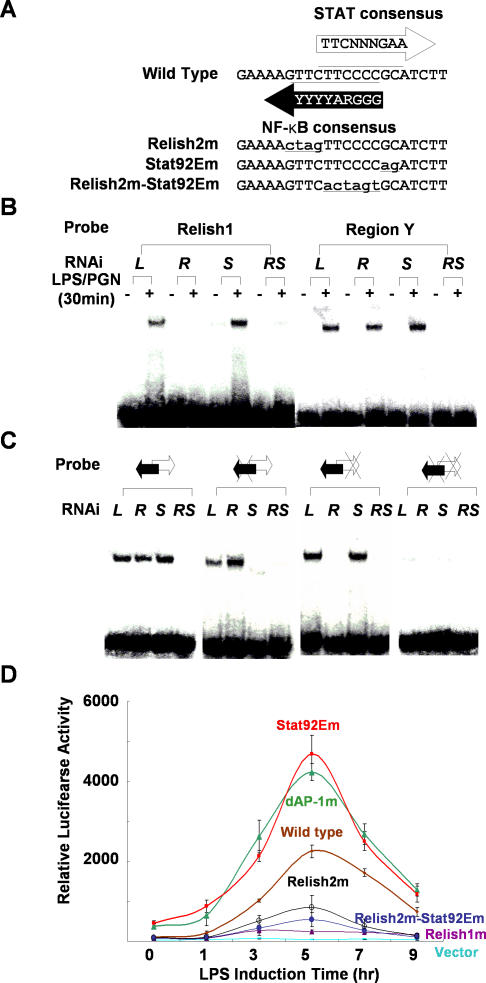
A Stat92E Binding Site on the *Attacin-A* Promoter Plays a Crucial Role in Down-Regulating *Attacin-A* (A) Region Y contains an NF-κB binding site and a STAT binding site. The NF-κB consensus and STAT consensus binding sequences are shown along with the wild-type sequence of region Y. The mutant forms of the NF-κB and/or the STAT binding sites of region Y are designated Relish2m, Stat92Em, and Relish2m-Stat92Em, respectively. The mutated sequences are shown in lower case and underlined. (B) Nuclear extracts of SL2 cells pre-incubated with dsRNA for *Luciferase* (L), *Relish* (R), *Stat92E* (S), or both *Relish* and *Stat92E* (RS) and treated with 10 μg/ml of LPS/PGN were assayed by EMSAs with ^32^P-labeled double-stranded oligonucleotide probes containing Relish1 or region Y. (C) EMSAs with probes containing the wild-type region Y (double arrow), a mutation in the Relish binding site (double arrow with X on the left), a mutation in the Stat92E binding site (double arrow with X on the right) or mutations in both binding sites (double arrow with double X). Black and white arrows indicate the Relish 2 and Stat92E binding sites, respectively, and the mutations are indicated by Xs. Nuclear extracts were as in (B). (D) The dAP-1 and Stat92E promoter elements are required for down-regulation of *Attacin-A*. SL2 cells transfected with each reporter under the control of a mutant version of the *Attacin-A* promoter as indicated were treated with 10 μg/ml LPS/PGN for the time indicated on the abscissa. The mean levels of the normalized luciferase activities are shown with standard deviations. These experiments were repeated at least three times independently.

To examine whether the activated form of Stat92E exerts its repressive role directly by binding to the promoter of *Attacin-A,* we examined the upstream regions of the *Attacin-A* genes of a number of *Drosophila* species to identify evolutionarily conserved Stat92E and other transcription factor binding motifs (Figure S2). Sequence alignment revealed several strongly conserved regions: in addition to the core promoter elements (TATA and initiator motifs), we identified a Relish-binding motif (−140 bp), a GATA motif (−130 bp), and a dAP-1–binding motif (−90 bp), along with a highly conserved region (region Y at −45 bp) upstream of the TATA box. Intriguingly, this region contains an Relish-binding motif [18] that overlaps with a sequence showing weak homology to the STAT consensus binding sequence [19] in the opposite strand (Figure 2A). To test for binding of these motifs by the corresponding transcription factors, we performed electrophoretic mobility shift assays (EMSAs) with a probe spanning region Y, and we compared the results with those obtained with a probe for the distal Relish-binding motif. LPS/PGN treatment of SL2 cells led to strong mobility shifts of both probes, and these were competed out by an excess of cold probe (unpublished data). Because the region Y probe contained both the Relish- and Stat92E-binding motifs, the addition of a specific antibody against one or other of these transcription factors resulted in supershifting only a portion of the shifted bands (Figure S3 and unpublished data). Therefore, we confirmed the identity of the protein(s) bound to each probe by repeating the EMSAs after depleting Relish or Stat92E, or both, by RNAi (Figure 2B). The LPS/PGN-induced mobility shift of the distal Relish probe (Relish1) was lost when Relish was depleted but not when Stat92E was depleted, whereas the shift of the region Y probe was only abolished when both Relish and Stat92E were depleted, indicating that both transcription factors bind to their putative binding sites in this probe. This interpretation was confirmed by showing that the LPS/PGN-induced mobility shift was not affected by mutations affecting only the Relish or the Stat92E binding sequence, but was abolished when both binding sequences were mutated (Figure 2C). Moreover the Stat92E mutant probe was not shifted in Relish-depleted extracts, and the Relish2 mutant probe was not shifted in Stat92E-depleted extracts. These results establish that region Y contains genuine Relish- and Stat92E-binding sites.

Although the putative GATA-binding sequence in the *Attacin-A* promoter is also strongly conserved (Figure S2B), *Drosophila* GATA homologs do not appear to play a major role in LPS/PGN-induced *Attacin-A* transcription, at least in SL2 cells, since depletion of the *Drosophila* GATA factor Serpent by RNAi had no discernable effect on LPS/PGN-induced *Attacin-A* transcription (unpublished data).

We also generated luciferase reporters under the control of the mutant versions of the *Attacin-A* promoter and examined LPS/PGN-induced luciferase activities after transient transfection of the reporters (Figure 2D). Mutation of either of the Relish-binding motifs inhibited transcription from the *Attacin-A* promoter such that no (Relish1 mutation) or only weak (Relish2 mutation) luciferase activity was detected. In contrast, mutation of the Stat92E- or dAP-1-binding motifs resulted in at least 3-fold higher luciferase activities than obtained with the wild-type promoter. This result, along with the result from EMSAs, demonstrates that the defects in the binding of Relish, Jra, and Stat92E to their binding motifs in the promoter result in altered *Attacin-A* transcription. Therefore, both Jra and Stat92E appear to down-regulate genes activated by Relish in response to pathogen-associated molecular patterns.

### Interdependence of Jra and Stat92E for Stable Binding to the Relish Target Promoter

**Figure 3:**
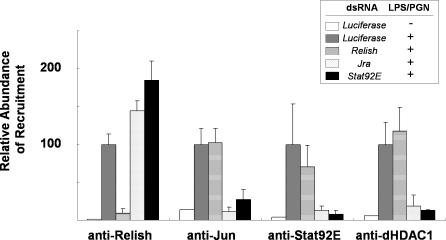
Synergistic Binding of Jra and Stat92E to the *Attacin-A* Promoter ChIP assays of the transcription factors indicated below using various mutants. SL2 cells were depleted of the transcripts by dsRNA treatment, as indicated in the top box, for 3 d. Then chromatin extracts were prepared before (−) or after 30 min (+) of LPS/PGN treatment. The amounts of *Attacin-A* promoter fragments co-precipitated with antibodies against the transcription factors indicated below the data were measured by real-time PCR. The levels were normalized by the input used in each ChIP assay and are shown with standard deviations. These experiments were repeated independently at least three times.

To test whether similar transcription factor binding occurs in a chromosomal context, we examined the recruitment of transcription factors by means of chromatin immunoprecipitation (ChIP) assays. These experiments showed that LPS/PGN treatment induced the synthesis and nuclear translocation of Relish, Jra, and Stat92E and their binding to the promoter (Figure 3). Relish knock-down reduced Relish without affecting the binding of the other transcription factor. On the other hand, depletion of Jra caused the loss not only of its own binding but also of that of Stat92E, and vice versa, indicating that Jra and Stat92E bind synergistically to the Relish target promoter. This co-occupancy of the promoter by Jra and Stat92E may elicit the repressive function of the transcription factors. As shown previously, dHDAC1 is also recruited to the *Attacin-A* promoter after LPS/PGN-treatment, and this also requires concurrent binding of Stat92E and Jra. Knock-down of EcR or Mad had no effect on recruitment of Relish, Jra, Stat92E, and dHDAC1 (unpublished data).

### Formation of a dHDAC1-Containing Complex with Jra, Stat92E, and the HMG Protein Dsp1

**Figure 4:**
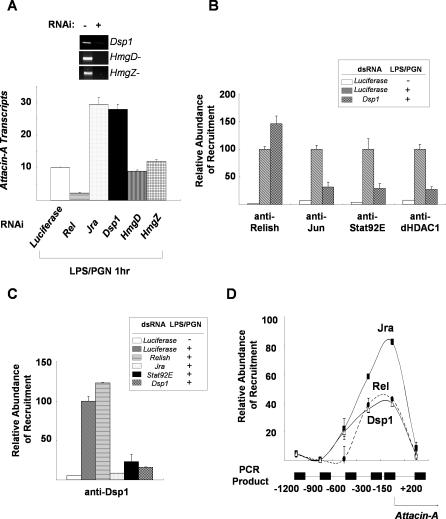
Dsp1 Plays a Crucial Role in the Interactions between Jra and Stat92E (A) Down-regulation of *Attacin-A* transcripts by HMG protein. Real-time PCR analysis showing *Attacin-A* transcript levels after 1 h of LPS/PGN treatment of SL2 cells depleted by RNAi of the transcription factors and HMG proteins indicated below the histograms. The levels were normalized with *RpL32* transcripts. The extents of depletion of the corresponding transcripts by RNAi are shown in the top panel. (B) Dsp1 is required for binding of Jra, Stat92E, and dHDAC1 to the *Attacin-A* promoter. SL2 cells were incubated with *Luciferase* or *Dsp1* dsRNA for three days, then used in ChIP assays with (+) and without (−) LPS/PGN treatment (10 μg/ml for 1 h). The amounts of *Attacin-A* promoter fragments co-precipitated with the antibodies were normalized for the input used in each assay and are shown with standard deviations. These experiments were repeated at least three times independently. (C) Requirement for Jra and Stat92E for recruitment of Dsp1 to the *Attacin-A* promoter. SL2 cells were depleted of the protein indicated on the right by RNAi, then used in ChIP assays before (−) and after (+) LPS/PGN treatment. The amounts of *Attacin-A* promoter co-precipitated with anti-Dsp1 antibody in the ChIP assays are shown with standard deviations. These experiments were repeated at least three times independently. (D) The amounts of chromatin fragments co-precipitated with anti-Jun (solid squares), anti-Dsp1 (open triangles) or anti-Relish (solid circles) antibodies in the indicated regions of the *Attacin-A* promoter were measured by real-time PCR in a Roche Lightcycler, and the averages and standard deviations of three independent experiments are plotted.

The recruitment of dHDAC1 to the promoter bound by both transcription factors suggested that some factor, such as an HMG protein, is required to mediate the interactions between the transcription factors and histone deacetylase. We therefore examined the requirement for *Drosophila* HMG proteins (Dsp1, HmgD, and HmgZ) for the Jra- and Stat92E-mediated down-regulation of *Attacin-A* transcription. Depletion of Dsp1 mimicked the effect of Jra knock-down, whereas knock-down of HmgD or HmgZ had no detectable effect (Figure 4A). In addition, depletion of Dsp1 prevented the LPS/PGN-induced binding of Jra, Stat92E, and dHDAC1 (Figure 4B). Interestingly, ChIP with antibody to Dsp1 revealed that Dsp1 was recruited to the *Attacin-A* promoter by LPS/PGN treatment, and this was completely dependent on Jra and Stat92E, suggesting that Dsp1 is specifically localized to the *Attacin-A* promoter region in response to LPS/PGN (Figure 4C). To test this idea, we performed ChIP assays with antibodies against Dsp1, Relish, and Jra, monitoring the upstream region of *Attacin-A* from −1.2 kb to the coding sequence. The amount of chromatin co-precipitated with anti-Dsp1, anti-Relish, or anti-Jun antibodies reached a peak in the region 150 base pairs (bp) upstream from the transcription initiation site where the canonical Relish and dAP-1 binding motifs are located (Figure 4D). Moreover we found a putative Dsp1 binding site (GAAAA) within this region (Figure S2). Therefore, Dsp1 appears to be required for the interaction between the transcription factors and histone deacetylase on the *Attacin-A* promoter

### Induction of the Jra/Stat92E/Dsp1 Complex

**Figure 5:**
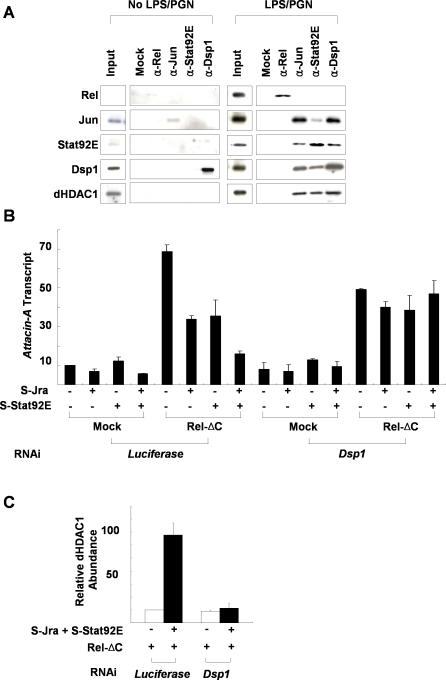
Dsp1 Interacts with Jra and Stat92E to Form a Repressosome Complex (A) Co-immunoprecipitation of Relish, Jra, Stat92E, and Dsp1. Nuclear extracts prepared from SL2 cells with (right panel) or without (left panel) LPS/PGN (10 μg/ml for 45 min) treatment were immunoprecipitated with the antibodies indicated at the top of the figure, and the amounts of the proteins in the pellets were measured by immunoblot analysis with the antibodies indicated on the left. For co-immunoprecipitation of Relish, Jra, and Stat92E, 10-μg aliquots of nuclear extracts were used, whereas for Dsp1, 3-μg aliquots were used. Five percent of the amount of nuclear extract used in each immunoprecipitation assay is shown as Input. (B) Regulation of *Attacin-A* transcription by ectopic expression of transcription factors. The N-terminal half of Relish that is competent as a transcriptional activator (Rel-ΔC), epitope-tagged Jra (S-Jra), and Stat92E (S-Stat92E) expression constructs were transfected into SL2 cells as indicated at the bottom of the figure. After induction of the recombinant proteins, the levels of *Attacin-A* transcripts relative to those of *RpL32* were measured by real-time PCR analysis in three independent experiments. (C) ChIP assays of the *Attacin-A* promoter with anti-dHDAC1 antibody. SL2 cells pretreated with *Luciferase* dsRNA (control) or *Dsp1* dsRNA (Dsp1-) were transfected with expression constructs for Rel-ΔC, S-Jra, and S-Stat92E as indicated at the bottom, and the average amounts of *Attacin-A* promoter fragments co-precipitated with anti-dHDAC1 antibody after induction of the transfected transcription factors were measured by real time PCR analysis in three independent experiments.

In order to prove that Jra, Stat92E, Dsp1, and dHDAC1 form a repressosome complex, we also examined the physical interactions between them. Immunoprecipitation experiments demonstrated that Jra, Stat92E, and Dsp1, but not Relish, were co-precipitated from LPS/PGN-treated SL2 cells in a dHDAC1-containing complex by antibody against any one of them (Figure 5A). These interactions do not appear to be mediated by DNA, because the addition of a high concentration of ethidium bromide to the extracts did not affect their co-immunoprecipitation (unpublished data). Similar immunoprecipitation experiments with nuclear extracts of non–LPS/PGN-treated SL2 cells brought down only trace amounts of the corresponding transcription factors. We conclude that LPS/PGN treatment induces activation and nuclear transport of Relish, as well as the formation of a complex containing Jra, Stat92E, Dsp1, and dHDAC1.

The formation of a dHDAC1-containing complex with Stat92E and Jra prompted us to examine the possibility that Jra, Stat92E, or Dsp1 are modified in some way during LPS/PGN signaling. However, we failed to detect any LPS/PGN-induced mobility shift of these factors by two-dimensional electrophoresis followed by Western analysis (unpublished data).

To investigate whether an increase in the levels of Jra, Stat92E, and Dsp1 upon LPS/PGN treatment is instead the major determinant of repressosome formation, we set up a system in which *Attacin-A* transcription was driven by overexpression of the Relish N-terminal domain (Rel-ΔC) without the need for LPS/PGN treatment. We found that in this system, ectopic overexpression of Jra and Stat92E down-regulated *Attacin-A* transcription (Figure 5B), and ChIP with antibody to dHDAC1 revealed that histone deacetylase was only recruited to the *Attacin-A* promoter when Jra and Stat92E were overexpressed (Figure 5C). In addition, the inhibitory effect of the exogenous Jra and Stat92E was also dependent on the presence of Dsp1. These results suggest that elevated concentrations of Jra, Stat92E, and Dsp1 lead to the formation of the repressosome.

### Displacement of Relish from the *Attacin-A* Promoter by a Repressosome Complex

**Figure 6:**
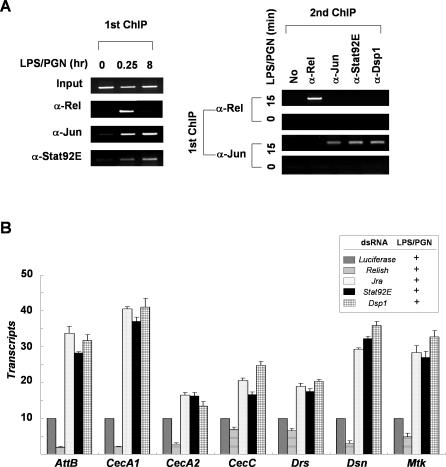
Relish Is Displaced from the *Attacin-A* Promoter by the Repressosome (A) Left panel: soluble chromatin extracts were prepared from SL2 cells with (15 min or 8 h) or without (0 min) LPS/PGN treatment, and immunoprecipitated with antibodies against Relish, Jun, or Stat92E as described in Figure 3. Right panel: double ChIP assays. The precipitates obtained from the first ChIP of cells with (15 min) or without LPS/PGN treatment were analyzed separately in a second ChIP with the antibodies indicated at the top (second ChIP). The amounts of *Attacin-A* promoter fragments co-precipitated with the indicated antibody are shown. (B) The transcript levels of each target gene are shown under *Luciferase, Relish, Jra, Stat92E,* or *Dsp1* knock-down conditions with LPS/PGN treatment (10 μg/ml; 1h). The averages and standard deviations of triplicates assays are shown.

To investigate the relationship between occupation of the *Attacin-A* promoter by the repressosome complex and by Relish, we examined the binding kinetics of these transcription factors to the promoter (Figure 6A). The ChIP results showed that both Relish and the repressosome complex were recruited to the *Attacin-A* promoter by 15 min after LPS/PGN treatment. Sustained incubation with LPS/PGN (8 h) resulted in loss of the Relish binding without loss of Jra and Stat92E binding. To examine whether Relish co-occupies the promoter with the repressosome complex during the early stage of LPS/PGN induction, we analyzed the chromatin fragments precipitated with antibody to Relish for the presence of the repressosome complex by means of a second ChIP (Figure 6A). Sequential ChIPs revealed that the Relish-bound *Attacin-A* promoter was devoid of Jra, Stat92E, and Dsp1, and that the Jra-associated *Attacin-A* promoter was devoid of Relish. Therefore, Relish and the Jra/Stat92E/Dsp1 repressosome complex occupy the *Attacin-A* promoter in a mutually exclusive fashion. These results indicate that Relish is displaced from the *Attacin-A* promoter by the repressosome complex, and that this results in the termination of transcriptional activation. Based on these observations, we postulate that at the outset of LPS/PGN-induced activation, only processed Relish is available to activate transcription; but as the newly synthesized and translocated Jra, Stat92E, and Dsp1 accumulate inside the nucleus, they form a repressosome complex and displace Relish from the promoter to terminate transcription.

The Jra/Stat92E/Dsp1/dHDAC1-mediated down-regulation of Relish-driven transcription does not appear to be limited to the *Attacin-A*. Among the seven additional antimicrobial peptide (AMP) genes *(Attacin-B, Cecropin A1, Cecropin A2, Cecropin C, Drosomycin, Drosocin,* and *Metchnikowin)* tested, four AMP genes *(Attacin-B, Cecropin A1, Drosocin,* and *Metchnikowin)* showed an identical pattern of regulation to *Attacin-A* (Figure 6B). Although the suppressive effect on the expression of other AMP genes was relatively small, the repressosome complex appears to down-regulate their expression in some degree. Considering that *Drosomycin* is activated mainly by Dif rather than Relish, this result suggests that the repressosome activity may vary depending on the types of NF-κB homologs present at the target gene promoters. Consistently, we found that both Jra and Stat92E binding sites as well as the NF-κB binding sequence exist on the promoter regions of these antimicrobial peptide genes (Figure S4). Therefore, the competitive binding of the Jra/Stat92E/Dsp1-containing repressosome complex to the Relish-binding site appears to be responsible for the termination of Relish-dependent gene transcription.

### Physiological Function of the Jra/Stat92E/Dsp1-Containing Repressosome Complex

In order to confirm the physiological relevance of the Jra/Stat92E/Dsp1 repressosome complex, we tested for defects in transcription of the Relish-dependent antimicrobial peptide genes during bacterial infection in mutant flies in which Stat92E, Jra, or Dsp1 levels were significantly reduced. To this end, we generated transgenic flies in which *Stat92E* RNAi was induced conditionally under the control of either *daughterless* or the minimal heat shock promoter. These mutant flies showed no obvious developmental defects; however quantitative reverse-transcriptase (RT)-PCR and Western blot analysis revealed that upon induction of the RNAi, the level of Stat92E declined significantly in the mutant flies (Figure S5A–S5D). The heterozygous Jra mutant flies and the homozygous Relish or Dsp1 mutant flies were viable, and Western blot analysis with the corresponding antibodies revealed a significant reduction (Jra) or almost complete loss (Relish and Dsp1) of the corresponding proteins in the mutant flies (Figure S5E and S5F).

**Figure 7:**
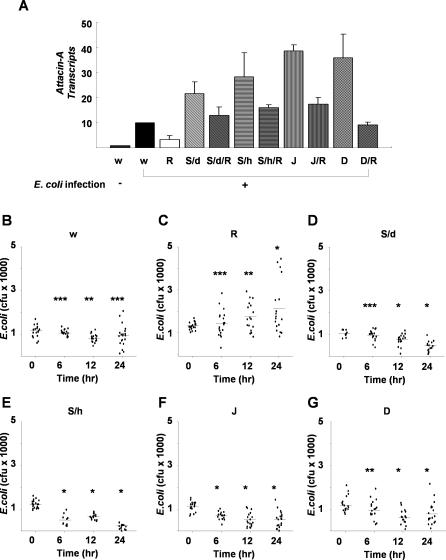
Up-Regulation of Relish Target Genes in Repressosome Mutant Flies during Bacterial Infection (A) Transcript levels of LPS/PGN-induced *Attacin-A* in mutant flies. Wild type *(w^1118^)* and mutant flies were infected with E. coli, and the levels of the *Attacin-A* transcript were measured by real time PCR analysis 24 h after infection. Total RNA from groups of five flies was pooled for the analysis. The averages and standard deviations of three independent assays are shown. *Attacin-A* was not expressed without bacterial infection in any of the mutants (unpublished data). Abbreviations for the mutant flies are as follows: w, *w^1118^;* R, *Rel/Rel;* S/d, *UAS-shStat92E/+; da-Gal4/+;* S/d/R*, UAS-shStat92E/+; da-Gal4/Rel;* S/h, *UAS-hs-shStat92E/UAS-hs-shStat92E;* S/h/R, *UAS-hs-shStat92E/+; Rel/+*; *J, Jra^1A109^/CyO*; *J/R, Jra^1A109^/+; Rel/+*; *D, Dsp1[EP355]/Dsp1[EP355]; D/R, Dsp1[EP355]/Y; Rel/+*. (B–G) Bacterial clearance assays. Wild type and mutants were injected with the same number of E. coli, and the number of live bacteria inside each injected fly was measured as described in Materials and Methods and represented by a dot on the graph. The bars represent mean of colony forming unit (cfu). The abbreviations used are as in (A). *p-*values were calculated by Student's *t-*test. *p < 0.01. **p < 0.05. ***p > 0.2.

In the absence of bacterial infection, all the mutant and wild-type flies appeared to be normal and did not make the *Attacin-A* transcript (unpublished data). Upon bacterial infection, *Attacin-A* transcription was induced more than 3–5-fold in the wild-type flies *(w^1118^)* but not in the Relish homozygotes *(Rel/Rel)*. Consistent with the in vitro result, the Stat92E RNAi mutants *(UAS-shStat92E/+; da-Gal4/+;* and *UAS-hs-shStat92E/UAS-hs-shStat92E),* the Jra heterozygotes *(Jra^1A109^/CyO,* and the Dsp1 homozygotes *(Dsp1[EP355]/Y)* contained several-fold higher levels of *Attacin-A* transcripts than bacterially infected wild type flies (Figure 7A and Figure S6), and a similar result was obtained from an analysis of *Drosocin* transcripts (unpublished data). This hyperactivation of *Attacin-A* transcription in the mutant flies was rescued by introducing a copy of the Relish mutant allele, indicating that Stat92E, Jra, and Dsp1 are required for the down-regulation of Relish during infection. Reflecting this result, the Relish mutants were defective in clearing the infecting bacteria, whereas the Stat92E, Jra, and Dsp1 mutants had even higher bacterial clearance activities than wild type flies did (Figure 7B–7G).

**Figure 8:**
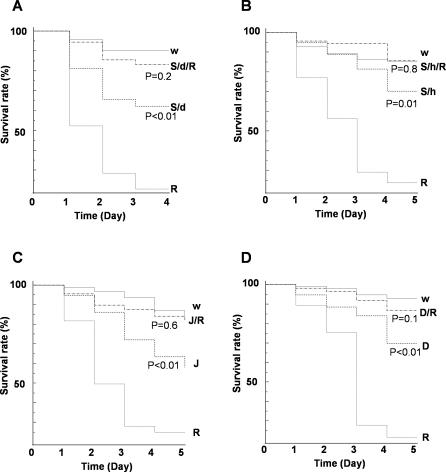
Lower Survival Rate Caused by Mis-Regulation of Repressosome Complex in Flies Survival of various mutant flies after bacterial infection. Three-d-old wild-type and mutant flies were infected with E. coli and their survival was measured each day after infection. Survival curves are plotted as Kaplan-Meier plots. Statistical significance is tested using log-rank analysis with MedCare software. The abbreviations used are as in Figure 7.

Continuous activation of NF-κB after clearance of infected pathogens in mammals usually causes damage and, in severe cases, septic injury [20,21]. Therefore, down-regulation of NF-κB target genes by a repressosome complex should play an important role in maintaining a proper balance between immune responses. To determine whether mis-regulated expression of Relish results in an excessive immune response that may be harmful to *Drosophila,* we examined the survival rates of these mutant flies after bacterial infection (Figure 8). Under conditions of bacterial infection that enabled most wild-type flies to survive but killed most Relish homozygotes within 4 d, the Stat92E RNAi mutant flies, the Jra heterozygotes, and the Dsp1 homozygotes all displayed reduced survival comparable to Relish heterozygotes (50% survived beyond 4 d). The increased mortality of the mutants appears to result from the immune response of the flies against the bacterial infection. No obvious survival defect was observed when phosphate-buffered saline (PBS) was injected instead of bacteria (Figure S7). Most importantly, combining Relish heterozygosity with mutations of Stat92E, Jra, or Dsp1 overcame the lethal consequence of bacterial infection rather than aggravating them. Hence, the increased vulnerability to infection of the Stat92E, Jra, and Dsp1 mutants is due to excessive activation of Relish target genes rather than to reduced activation of Stat92E, Jra, or Dsp1 target genes. Therefore, we conclude that down-regulation of NF-κB by the Jra/Stat92E/Dsp1-containing repressosome complex also occurs under physiological conditions and plays an important physiological role.

## Discussion

An excessive inflammatory response is harmful to the host; it can even be fatal [22–24] and must be prevented by negative-feedback mechanisms. Several such mechanisms, which mainly function by reducing NF-κB activation, have been identified [21–25], but little is known of their underlying mechanisms. Our demonstration that AP-1 and STAT are directly involved in down-regulating NF-κB illustrates the context-dependent use of transcription factors to achieve fine control of gene expression.

Ligand-induced conformational changes have been implicated in switching nuclear receptors from activators to repressors; however, it is not clear what makes other types of transcription factors act sometimes as activators and other times as repressors. We have shown above that a specific HMG protein functions as a core element nucleating the assembly of a repressor complex of AP-1, STAT, and HDAC1. The mammalian homolog of Dsp1, HMGB1, binds to a negative regulatory element adjacent to the NF-κB binding site in the interferon-beta enhancer [26], and Dsp1 is a co-repressor that converts Dorsal from activator to repressor by binding to G(A) motifs adjacent to Dorsal binding sites [27–-29]. Dsp1 is also required for correct expression of homeotic genes, and to recruit polycomb group proteins to polycomb and trithorax response elements [30]. Evidently, the role of Dsp1 is to facilitate the formation of repressor complexes on NF-κB–dependent promoters.

It is noteworthy that a different HMG protein, HMG-Y/I, forms repressosomes with the transcription factors NF-Y and BTEB-1 that represses transcription of a growth hormone receptor gene by recruiting the histone deacetylase complex [31]. This suggests that different HMG proteins associate with different transcription factors to regulate particular groups of genes [32,33]. Thus, the role of Dsp1 in forming a repressosome complex with AP-1 and STAT that inhibits specific types of NF-κB target promoters is one instance of an evolutionarily conserved mechanism. It may be a key NF-κB pathway regulatory mechanism, assuring an appropriate immune response.

It is well established that JNK and JAK/STAT signaling are involved in innate *Drosophila* immune responses [11,12,34,35]. Because the JNK pathway is primarily involved in cellular processes such as phagocytosis, wound healing, melanization, and defense against extracellular pathogens [36–39], our observation of increased lethality of the Jra mutant upon bacterial infection is unlikely to be due exclusively to malfunction of the repressosome complex. Nevertheless, the repressosome complex may well be an important component of *Drosophila* immune responses, since many Stat92E and Dsp1 mutant flies die upon bacterial infection, and this enhanced lethality was reversed by reducing Relish copy number. We propose that JNK participates in cellular immune responses and also forms a repressosome complex with Stat92E, Dsp1, and dHDAC1 that restricts the production of antimicrobial peptides.

Recently, Delaney et al. [40] have claimed that JNK is required for the synthesis of antimicrobial peptide genes upon bacterial infection of *Drosophila*. This claim conflicts with our results and also with a report that JNK activity is reduced by activation of NF-κB [11]. In the latter work [11], it was shown that expression of *Attacin-A* was enhanced by knock-down of JNK. The discrepancy between Delaney's result and ours may be due to differences between the mutants or methods used in the two studies. First, the extent of knock-down of gene activities differed: in our study, we reduced the expression of various genes (Jra, dJNK, and Stat92E) by conditional knock-down or by reducing copy number (Jra), whereas Delaney et al. clonally deleted dJNK and Jra and overexpressed a JNK inhibitor (Puc), and these procedures may have affected an essential function of the JNK pathway required for Relish-mediated transcriptional activation. Another possible explanation of the discrepancy derives from the use of different Jra alleles in the two studies. Unlike flies carrying the *Jra^1A109^/CyO* allele used by us, the heterozygous mutant flies carrying the *Jra^1^/CyO* allele used by Delaney et al. did not show any defect in Relish-dependent transcriptional activation of AMP genes even when they were examined under our experimental conditions. According to FlyBase, the truncated protein is stopped at the 177th amino acid in the *Jra^1^/CyO* allele and at the 72nd amino acid in the *Jra^1A109^/CyO* allele. The reason of the different lesions between two alleles needs further investigation.

With regard to the role of the JAK/STAT pathway in innate immunity in *Drosophila,* JAK/STAT pathway mutants have only been reported to have defects in antiviral responses and hemocyte function [34,35]. Though there have been efforts to identify the role of JAK/STAT in the innate immune response by genome-wide RNAi screening [41,42], the basis of the precise regulation of immune responses by this essential transcription factor remains unclear. Ours is the first evidence, to our knowledge, that the JAK/STAT pathway regulates the synthesis of antimicrobial peptide genes in *Drosophila*. Intriguingly, Agaisse and co-workers [34] found that expression of *Drosomycin* was enhanced upon bacterial infection in a loss-of- function mutant of Hopscotch. This finding is supported by our observation that functional Stat92E negatively regulates the synthesis of Relish-dependent antimicrobial peptides by forming a complex with Jra and Dsp1.

## Materials and Methods

### RNAi experiments.

Double-stranded RNA (dsRNA) was prepared as described previously [12]. *Drosophila* SL2 cells (1 × 10^6^; CRL-1963, American Type Culture Collection) were washed with serum-free *Drosophila* medium (Welgene; http://www.welgene.com) and treated with specific dsRNAs (20 μg) for 3 h. Serum was added back to the culture medium to 10% final concentration and the cells were incubated for an additional 72 h. The primers used for making the dsRNAs are listed in Table S1. The efficiency of knock-down in each RNAi experiment was confirmed by RT-PCR or Western blotting.

### RNA analysis.

Total RNA was isolated from SL2 cells with Trizol reagent (Invitrogen; http://www.invitrogen.com) and used for cDNA synthesis with Superscript II reverse transcriptase (Invitrogen). The abundance of transcripts in each cDNA sample was measured by real-time PCR using a Lightcycler (Roche; http://www.roche.com). The PCR reactions contained 1 × SYBR Green mix (Applied Biosystems; http://www.appliedbiosystems.com) or 1 × Taqman probe (Applied Biosystems), 10 pmol of forward and reverse primers, and cDNA corresponding to 0.1 μg of total RNA. The reactions were subjected to 40 cycles of PCR amplification (95 °C for 10 s, 55 °C for 20 s, and 72 °C for 30 s), and analyzed with Lightcycler Software 4 (Roche). All results were normalized to the level of *RpL32* mRNA in each sample. The real-time PCR analyses were repeated at least three times independently, and the means and standard deviations were calculated. The primers used in the real time PCR analyses are listed in Table S1.

### ChIP.

ChIP experiments were performed as described previously [12]. For most of the ChIP experiments in this study, 500–1,000-bp-length chromatin fragments were used except for the ChIP used to scan the *Attacin-A* promoter region, in which 200–300-bp fragments were used (Figure 4D). The antibodies were either raised in rats using recombinant Relish [from 270 amino acids (aa) to 540 aa], Stat92E (from 1 aa to 267 aa), Dsp1 (from 1 aa to 393 aa), and dHDAC1 (from 114 aa to 504 aa), or purchased from Santa Cruz Biotechnology (anti-Jun rabbit antibody; http://www.scbt.com). Dissociated DNA fragments were recovered with a QIAquick purification kit (Qiagen; http://www.qiagen.com) and subjected to 30 cycles of PCR (94 °C for 1 min, 55 °C for 1 min, and 72 °C for 1 min, followed by one cycle of 72 °C for 5 min) with specific primers. For ChIP-ChIP experiments, the complexes immunoprecipitated with the first antibody were eluted from the antibody beads by incubation with 10 mM dithiothreitol (DTT) at 37 °C for 30 min, diluted 1:50 in buffer (1% Triton X-100, 2 mM ethylene diamine tetraacetic acid [EDTA], 150 mM NaCl, 20 mM Tris, pH 8.0), and immunoprecipitated with the second antibody [43]. All subsequent procedures were essentially as for the primary ChIPs. The precipitated chromatin fragments were quantitated by real-time PCR with a Lightcycler with the primers listed in Table S1. The amount of precipitated chromatin measured in each PCR was normalized with the amount of chromatin present in the input of each immunoprecipitation. All experiments were repeated at least three times.

### Analysis of proteins.

Whole-cell extracts of SL2 cells were prepared in lysis buffer (20 mM Tris, pH 7.6, 150 mM NaCl, 10% glycerol, 1% Triton X-100, 25 mM β-glycerophosphate, 1 mM DTT, 2 mM EDTA, and protease inhibitors). Aliquots of the extracts (30 μg) were transferred to nitrocellulose membranes after resolving them by SDS-PAGE and probed with anti-Relish (1:1000), anti-Stat92E (1:1000), anti-Dsp1 (1:500), anti-dHDAC1 (1:1000), anti-Jun (1:1000), and anti-γ tubulin (1:1000). The antibodies were diluted in TBST (40 mM Tris, pH 7.4, 200 mM NaCl, 0.1% Tween20) by the factors shown in the parentheses, and the complexes were visualized with an ECL plus Detection System (Amersham Biosciences; http://www.amersham.com) after reaction with appropriate peroxidase-conjugated secondary antibody (1:10000; Sigma; http://www.sigmaaldrich.com). For co-immunoprecipitation, nuclear extracts were prepared from SL2 cells incubated with or without 10 μg/ml LPS/PGN (Sigma) as described previously [44]. After pre-clearing with Protein G beads (Invitrogen), antibody (5 μg) was added and the mixtures were incubated at 4 °C for 1 h followed by the addition of 50 μl of a 50% slurry of Protein G beads. Complexes were eluted with SDS loading buffer (50 mM Tris, pH. 6.8, 2% SDS, 0.1% bromophenol blue, 10% glycerol and 100 mM DTT), loaded on 10% SDS-PAGE gels and analyzed by Western blotting.

### Promoter alignment.

The *Attacin-A* sequences of five *Drosophila* species were obtained from the UCSC genome database and their promoter regions (from −2,000 bp to +1000 bp around the transcription initiation site) were retrieved. Putative binding sequences for each transcription factor were identified using the Transfac Professional 7.3 program (Biobase; http://www.biobase-international.com/), and sequences were aligned with vector NTI (Informax; http://www.informax.com). The default alignment parameters (gap opening penalty: 15; gap extension penalty: 6.66; gap separation penalty range: 8; score matrix: swgapdnamt) were used for alignment.

### Plasmid construction.

The wild-type pGL3-AttA plasmid was constructed by cloning the PCR-amplified sequence −2,400 to +32 of the *Attacin-A* promoter into *Xho*I*/Hind*III-digested pGL3 Basic vector (Promega; http://www.promega.com). A series of plasmids containing various mutations of the *Attacin-A* promoter were constructed from the pGL3-AttA wild-type plasmid. To generate pGL3-AttA Relish1m with a mutant Relish-binding site 1, we performed site-directed mutagenesis using a QuikChange Site-Directed Mutagenesis Kit (Stratagene; http://www.stratagene.com). To construct other mutant promoters, we introduced a suitable restriction site to mutate the transcription factor binding sequence. To this end we generated both upstream and downstream fragments of the transcription factor binding sites by PCR using specific primers in which the transcription factor binding sequence was replaced by a restriction enzyme recognition sequence. These fragments were digested with restriction enzyme and ligated to pGL3 Basic vector to generate a luciferase reporter construct under the control of the mutant promoter. pGL3-AttA dAP-1m, pGL3-AttA Relish2m-Stat92Em, pGL3-AttA Stat92Em and pGL3-AttA Relish2m reporters were constructed by replacing the relevant target sequences with *Eco*RI, *Spe*I, *Bgl*II, and *Spe*I sites, respectively. All the mutations introduced were confirmed by sequencing. The primers for these constructs are listed in Table S1.

### Reporter gene analysis.

Promoter constructs (100 ng) were transfected into SL2 cells (1 × 10^6^) using Cellfectin Reagent (Invitrogen). After 2 d, the transfected cells were treated with LPS/PGN (10 μg/ml) for various times and lysed with lysis solution (Tropix; http://www.appliedbiosystems.com/tropix). Firefly luciferase and β-galactosidase activities were analyzed with the dual-light luciferase assay system (Tropix) on an Infinite 200 instrument (Tecan; http://www.tecan.com). To normalize transfection efficiencies, a CMV-lacZ construct (20 ng) was co-transfected with each mutant reporter construct. Each reaction was assayed in duplicate, and the reporter analysis was repeated a minimum of three times independently.

### EMSAs.

EMSA experiments were performed as described previously [12]. To prepare SL2 nuclear extracts depleted for a given transcription factor, the cells (1 × 10^6^) were pre-treated with dsRNA for *Relish, Stat92E,* or both; 20 μg each [or *Luciferase* as a control]. After three days, the cells (3 × 10^7^) were lysed with 100 μl of NE buffer #1 (10 mM HEPES, pH 7.9, 10 mM KCl, 0.1 mM EDTA, 0.1 mM ethylene glycol tetra acetic acid [EGTA], 1 mM DTT, and protease inhibitors) for 10 min at 4 °C; 6 μl of 10% IGEPAL CA-630 was added and the suspension was centrifuged at 12,000*g* for 15 min to pellet nuclei. The supernatant was removed, and the pellet was resuspended in 50 μl of NE buffer #2 (20 mM HEPES, pH 7.9, 0.4 M NaCl, 1 mM EDTA, 1 mM EGTA, 10% glycerol, 1 mM DTT, and protease inhibitors). After further centrifugation at 12,000*g* for 20 min, the supernatant was obtained. To make the probes, 26-bp oligonucleotides containing the transcription binding sites were labeled with [γ-^32^P] ATP (Amersham Bioscience). The sequences of the oligonucleotides used are listed in Table S1. 10-μg samples of nuclear extract in a final volume of 20 μl EMSA binding buffer (100 mM NaCl, 15 mM HEPES, pH 7.5, 0.75 mM EDTA, 1 mM DTT, 1 μg poly dI-dC, and 8% glycerol) were chilled on ice for 15 min and then incubated for 30 min at room temperature with labeled probe. After addition of sucrose loading solution (40% sucrose, 0.25% bromophenol blue, and 0.25% xylene cyanol), the samples were loaded onto a 4% native polyacrylamide gel and run at 200 V for 80 min. The dried gel was exposed on an image plate and analyzed with a Cyclon Phosphor Screen (Packard; http://www.perkinelmer.com).

### Ectopic expression.

The expression construct for the N-terminal half of Relish was obtained from J. M. Park (Massachusetts General Hospital, United States). The full length Jra and Stat92E coding regions were cloned into the SRT-tagged SL2 cell expression vector, pSRT-MK33 [45]. The expression constructs (2 μg) were transfected into SL2 cells (3 × 10^6^), pre-treated with the corresponding dsRNA (*Dsp1* and *Luciferase* [control]; 20 μg each), and incubated for 2 d, then incubated with 0.7mM CuSO_4_ for 6 h to induce the production of recombinant proteins. The level of recombinant protein expression was analyzed by Western blotting with SRT monoclonal antibody (Daeil; http://www.daeil21.com/).

### Fly strains.

Transgenic Stat92E RNAi fly lines were obtained using an inducible RNAi method [46]. To construct the *Stat92E* RNAi element, two *Stat92E* fragments (one from 287–1,418 nucleotides (nt) including an intron and the other from 986–487 nt in the opposite direction) were amplified with the specific primers listed in Table S1 and ligated together into *Eco*RI/*Not*I-digested pBluescript vector (Stratagene) to yield pBS-shSTATi. The *Eco*RI/*Not*I fragment of pBS-shSTATi was inserted into *Eco*RI*/Not*I-digested pUAST P element transformation vector to yield pUAST-shSTATi. Germline transformation of *Drosophila* embryos with pUAST-shSTATi and identification of the P-element–integrated chromosome in each transgenic line was carried out as described previously [47]. A transgenic line with pUAST-shSTATi on the X chromosome was named *UAS-shStat92E,* and used in the analysis.

To activate transcription of the hairpin-encoding transgene, flies carrying a copy of both *UAS-shStat92E* and a *da*-*Gal4* driver were generated by crossing homozygous *UAS-shStat92E* female flies with homozygous male flies carrying the *da*-*Gal4* driver (which induces strong and ubiquitous expression of Gal4 protein) on the third chromosome (gift from B. Lemaitre, Centre National de la Recherche Scientifique, France). We also tried to generate Stat92E knocked-down transgenic flies controlled by the *hs-Gal4* driver (*UAS-shStat92E/+; hs-Gal4/+*). However, the shStat92E RNA expression in this mutant was too strong, and most of the flies died before infection, probably because of defects caused by the lack of Stat92E-dependent responses. Because the minimal heat shock promoter used in the *UAS-shStat92E* flies can be activated by heat shock treatment as shown in Figure S5, we exposed the *UAS-shStat92E* flies to heat shock (37 °C) three times a day to induce the Stat92E knock-down condition. To examine the effect of Stat92E knock-down in Relish heterozygotes, a Rel^E20^ homozygote was crossed with *UAS-shStat92E* homozygous flies to generate *UAS-shStat92E/+; Rel/+* double heterozygous progeny. *UAS-shStat92E/+; Rel/+* double heterozygous females were crossed with *da-Gal4* homozygous males to generate *UAS-shStat92E/+; Rel/da-Gal4* triple heterozygous progeny or exposed to heat shock to generate *UAS-hs-shStat92E/+; Rel/+* mutants. Knock-down of Stat92E expression in the transgenic flies was confirmed by real-time PCR analysis and immunoblotting with Stat92E antibody.

Jra mutants flies *(Jra^1A109^/CyO)* were obtained from D. Bohmann (University of Rochester Medical Center, United States). Because the Jra mutation was homozygous-lethal, Jra heterozygotes *(Jra^1A109^/CyO)* were crossed with Rel homozygotes *(Rel/Rel)* to generate *Jra^1A109^/+; Rel/+* flies. Dsp1 mutant flies *(Dsp1[EP355]/Dsp1[EP355])* were obtained from the Bloomington stock center. A Dsp1 homozygous female *(Dsp1[EP355]/Dsp1[EP355])* was crossed with a Rel homozygous male *(Rel/Rel)* to generate *Dsp1[EP355]/Y; Rel/+* male flies. Knock-down of Jra or Dsp1 expression in the transgenic flies was confirmed by Western blotting with Jun or Dsp1 antibody.

### Infection experiments.

The systemic response was triggered by pricking adult flies in the thorax with a thin tungsten needle dipped in a concentrated culture of *Escherichia coli,* or by injecting an E. coli suspension into adult flies with a pulled glass needle using a Picospritzer III injector (Parker Hannifin; http://www.parker.com). The glass needle was placed on the ventrolateral surface of the anterior abdomen as previously described [48]. For infection with bacteria, 3- to 4-d-old adult flies (15 males and 15 females) were anesthetized with CO_2_ and injected with a concentrated E. coli culture resuspended (1–5 nl; optical density = 200) in phosphate-buffered saline (137 mM NaCl, 2.7 mM KCl, 10 mM Na_2_HPO_4_, 2 mM KH_2_PO_4_). To study survival after infection, the injected flies were kept at 25 and the number of surviving flies was scored at 1-d intervals. Survival curves were plotted as Kaplan-Meier plots. Statistical significance was tested using log-rank analysis with MedCare software (http://www.medcare.be). To study bacterial clearance, flies after infection were homogenized in 100 μl of LB containing 1% Triton X-100 with a small pestle, and the homogenate was assayed by plating on LB-agar. These experiments were repeated at least four times.

## Supporting Information

Figure S1 Down-Regulation of *Attacin-A* Transcripts by *Hopscotch* and *Stat92E*
Levels of *Attacin-A* transcripts in SL2 cells treated with dsRNA for *Luciferase* (solid diamonds), *Hopscotch* (solid squares), *Stat92E* (open triangles), and *Relish* (open circles) are shown along with the time after LPS/PGN (10 μg/ml) treatment. The degree of depletion of the corresponding transcript by RNAi is shown in the bottom panel.
(659 KB TIF)

Figure S2 Alignment of the *Attacin-A* Promoter Regions of *Drosophila* Species (A) The upstream regions of *Attacin-A* genes of different *Drosophila* species have evolutionarily conserved transcription factor binding motifs. The alignment was performed using vector NTI (Informax). The absolute complexity of the *y-*axis was calculated as the sum of all pairwise residue substitution scores at a given alignment position normalized by the number of pairs in the alignment. A higher *y* value indicates higher sequence conservation. The *x-*axis indicates the distance from the start site on the *Attacin-A* promoter. The transcription factors are listed at the top.
(B) The promoter sequences of the *Attacin-A* genes of five *Drosophila* species that have diverged for at most 60 My were aligned with vector NTI (Informax) and visualized with the Box Shade 3.21 program at http://www.ch.embnet.org. The evolutionarily conserved sequence motifs are marked at the top. Sequences completely conserved are shown in black and sequences more than 80% conserved are shown in gray. The relative distances of the sequences from the transcription initiation site are indicated.
(2.1 MB TIF)

Figure S3 Supershift Assay Nuclear extracts of SL2 cells treated with or without 10 μg/ml of LPS/PGN were assayed by EMSAs with ^32^P-labeled double-stranded oligonucleotide probes containing region Y with or without anti-Stat92E antibody.
(734 KB TIF)

Figure S4 Putative Binding Sites of the Promoter Regions of AMP Genes The putative binding sequence of each transcription factor was assigned using the Transfac professional 7.3 program (Biobase). The putative transcription factor binding sites of the antimicrobial peptide genes of D. melanogaster are marked.
(613 KB TIF)

Figure S5 Knock-Down of Each Target Gene in Mutants (A) The levels of *Stat92E* transcripts (left panel) in wild-type *(w^1118^)* and STAT mutant *(UAS-shStat92E* or *UAS-shStat92E; da-Gal4)* flies before (−) and 3 h after (+) infection with E. coli were measured by RT-PCR. Levels of *RpL32* transcripts are shown as loading controls.
(B) The expression of Stat92E protein was measured by Western blotting in wild-type *(w^1118^)* and STAT mutant *(UAS-shStat92E; da-Gal4)* flies before (−) and after (+) infection. Levels of tubulin are shown as loading controls.
(C) The expression of a hairpin-encoding Stat92E transgene was measured in wild-type *(w^1118^)* and STAT mutant *(UAS-shStat92E)* flies before (−) and after (+) heat shock by RT-PCR. Levels of *RpL32* transcripts are shown as loading controls.
(D) The expression of Stat92E protein was measured by Western blotting in wild-type *(w^1118^)* and STAT mutant *(UAS-shStat92E)* flies before (−) and after (+) heat shock. Levels of tubulin are shown as loading controls.
(E) The expression of Jra protein was measured in wild-type *(w^1118^)* and Jra heterozygous mutant *(Jra^1A109^/CyO)* flies by Western blotting. Levels of tubulin are shown as loading controls.
(F) The expression of Dsp1 protein was measured in wild-type *(w^1118^)* and Dsp1 homozygous mutant *(Dsp1[EP355]/Y)* flies by Western blotting. Levels of tubulin are shown as loading controls.
(898 KB TIF)

Figure S6 Expression of *Attacin-A* upon Bacterial Infection In Vivo Wild-type *(w^1118^)* and Stat92E mutant flies *(UAS-shStat92E/+; da-Gal4/+)* were infected with E. coli, and the levels of the *Attacin-A* transcript were measured by real-time PCR analysis after infection. Total RNA from groups of five flies was pooled for the analysis. The averages and standard deviations of three independent assays are shown.
(521 KB TIF)

Figure S7 Survival Rate of Each Mutant upon PBS Survival of various mutant flies after PBS injection. Three-d-old wild-type and mutant flies were injected with PBS, and their survival was measured each day after injection. Survival curves are plotted as Kaplan-Meier plots. Statistical significance is tested using log-rank analysis with MedCare software. The survival curve of each mutant had a statistical significance (*p* > 0.2)
(544 KB TIF)

Table S1 Oligonucleotides Sequences Used in This Study (90 KB DOC)

## Abbreviations

AMP: antimicrobial peptide
ChIP: chromatin immunoprecipitation
Dsp: dorsal switch protein
EMSA: electrophoretic mobility shift assay
LPS/PGN: lipopolysaccharide/peptidoglycan
RNAi: RNA interference
