# Supplementary material for: Down-Regulation of NF-κB Target Genes by the AP-1 and STAT Complex during the Innate Immune Response in Drosophila
Source: PLoS Biol. 2007 Sep 4;5(9):e238. doi: 10.1371/journal.pbio.0050238 (PMC1964775; doi:10.1371/journal.pbio.0050238)
Supplement: Supplementary file 1 [file pbio.0050238.st001.doc]

**Table S1. Oligonucleotides sequences used in this study**

| **Name** | **Gene** | **Direction^a^** | **Sequence (5'-3')** |
| --- | --- | --- | --- |
| **dsRNA Primers** | | | |
| dsR-1 | Hopscotch | F | TTAGATATTGCAAATGGCAT |
|  |  | R | AATTTAGTTTAGTTTAGTTC |
| dsR-2 | STAT92E | F | AGGGCTCCCTGGATGATATC |
|  |  | R | TGCAATGTGCTCTTCACATA |
| dsR-3 | Jra | F | CCCGAATTCATGAAAACCCCCGTTTCC |
|  |  | R | CCCGGATCCTTGGTCTGTCGAGTTCGG |
| dsR-4 | Relish | F | CCCGGATCCTCCGCGCCCAGCCATATAA |
|  |  | R | CCCGAATTCAGTCCGAGGAGATGGACAT |
| dsR-5 | Dsp1 | F | CCCCTCGAGATGGAACACTTTCATCAA |
|  |  | R | CCCAAGCTTTTGGTTCTCGTCATCATC |
| dsR-6 | EcR | F | CCCGGATCCCGAGTGGACAACCCACGT |
|  |  | R | CCCAAGCTTGGCATGCTGGGGCGGCTC |
| dsR-7 | MAD | F | CCCACTAGTATGGACACCGACGATGTG |
|  |  | R | CCCACTAGTTCAGGATACCGAACTAATTGC |
| dsR-8 | HMG D | F | CGGCCCGGATCCATGTCTGATAAGCCAAAA |
|  |  | R | CGGCCCAAGCTTCTCGCTCTCATCATCGTC |
| dsR-9 | HMD Z | F | CGGCCCGGATCCATGAGTGGAGATCGCCCG |
|  |  | R | CGGCCCAAGCTTCTCGCTCTCGTCCTCCTC |
| dsR-10 | Serpent | F | ATCCGCCCAGCCATCATA |
|  |  | R | TAGCCGTCCTCCAAGGAG |
|  |  |  |  |
| **RT-PCR Primers** | | | |
| R-1 | Attacin-A | F | AGGTTCCTTAACCTCCAATC |
|  |  | R | CATGACCAGCATTGTTGTAG |
| R-2 | Attacin-B | F | GGGTAATATTTAACCGAAGT |
|  |  | R | GTGCTAATCTCTGGTCATC |
| R-3 | Cecropin A1 | F | ATGAACTTCTACAACATCTT |
|  |  | R | GGCAGTTGCGGCGACATTGG |
| R-4 | Cecropin A2 | F | ATTAGATAGTCATCGTGGTT |
|  |  | R | GTGTTGGTCAGCACACT |
| R-5 | Cecropin C | F | TGTAAGCTAGTTTATTTCTATGG |
|  |  | R | GATGAGCCTTTAATGTCC |
| R-6 | Drosomycin | F | CTTGTTCGCCCTCTTCGCTGTC |
|  |  | R | AGCACTTCAGACTGGGGCTGCA |
| R-7 | Drosocin | F | CCATCGTTTTCCTGCTGCTTGC |
|  |  | R | GTCAGGTGATCCTCGATGGCCA |
| R-8 | Metchnikowin | F | GCATCAATCAATTCCCGCCACC |
|  |  | R | CGGCCTCGTATCGAAAATGGG |
| R-9 | Puckered | F | CATGTGGCTAGCAATTTGA |
|  |  | R | GTCTTCGAAAAACGTACAGC |
| R-10 | CG15097 | F | TTCTGCCCGTGAAATCCAAT |
|  |  | R | GATTCTGGTGATGCGCCTC |
| R-11 | RpL32 | F | CAGTCGGATCGATATGCTAAGCTGT |
|  |  | R | TAACCGATGTTGGGCATCAGATACT |
|  |  |  |  |
| **Primers used for chromatin immunoprecipitation** | | | |
| C-1 | Attacin-A | F | GGCCAATGGTTTCGAGTTC |
|  |  | R | ATTGGGCGATGACCAGAG |
| C-2 | Attacin-A | F | CACAAGCAAACAGCAGAAGTAAAC |
|  |  | R | CATCGGTTTTATACCTCAAGATGC |
| C-3 | Attacin-A | F | ACGCTGACAGAGGTTCTG |
|  |  | R | AGCGAAATTCCCCGCTCA |
| C-4 | Attacin-A | F | AAAGTTTCCGCTCTCCTC |
|  |  | R | TTCTGGGGGAGCCGGAAA |
| C-5 | Attacin-A | F | CTGTTCTATAGCACGTTC |
|  |  | R | CATATGCAATTATATACGAA |
| C-6 | Attacin-A | F | TGTGCATACCGCGGAGAA |
|  |  | R | ACCCCTCATTGTGTCACA |
|  |  |  |  |
| **Primers used for reporter construction** | | | |
| Wild Type | Attacin-A | F | CCCCTCGAGTAATGGCTCACACGTGCCC |
|  |  | R | CCCAAGCTTGTTGCTGAACTGGATTGCTGG |
| Relish1m | Attacin-A | F | CCATCACATCTGAGCGACGAATTTCGCTTTGATAAGGC |
|  |  | R | GCCTTATCAAAGCGAAATTCGTCGCTCAGATGTGATGG |
| dAP-1m | Attacin-A | F | GCGGAATTCTGTCAATCATCAGAAAAGTTC |
|  |  | R | ATAGAATTCATCTGATTGCCGATCTCGGC |
| Relish2m-Stat92Em | Attacin-A | F | GCGAGATCTTGAGGTATAAAACCGATGCA |
|  |  | R | ATAACTAGTGAACTTTTCTGATGATTGAC |
| Relish2m | Attacin-A | F | GCGACTAGTTCCCCGCATCTTGAGGTATA |
|  |  | R | ATAACTAGTTTTCTGATGATTGACATGAT |
| Stat92Em | Attacin-A | F | GCGAGATCTTGAGGTATAAAACCGATGCA |
|  |  | R | ATAAGATCTGGGGAAGAACTTTTCTGATG |
|  |  |  |  |
| **pUAST-shStat92Ei Primers** | | | |
| U-sense | Stat92E | F | CCCGAATTCCCGTTGAGCTGTACACGC |
|  |  | R | CCCGGATCCACACGGATCTGGAAATCG |
| U-antisense | Stat92E | F | CCCGGATCCTGTTCAAGCAGATGTGTG |
|  |  | R | CCCGCGGCCGCCAGGGCATAGGACCTATC |
|  |  |  |  |
| **EMSA probes** | | | |
| Relish2-Stat92E WT | Attacin-A |  | GAAAAGTTCTTCCCCGCATCTTGAGG |
| Relish2m-Stat92Em | Attacin-A |  | GAAAAGTTCGAATTCGCATCTTGAGG |
| Relish2m | Attacin-A |  | GAAAACTAGTTCCCCGCATCTTGAGG |
| Stat92Em | Attacin-A |  | GAAAAGTTCTTCCCCAGATCTTGAGG |

^a^ F: Forward, R: Reverse
